# Impact of Smoking Status on Perioperative Morbidity, Mortality, and Long-Term Survival Following Transthoracic Esophagectomy for Esophageal Cancer

**DOI:** 10.1245/s10434-021-09720-6

**Published:** 2021-03-03

**Authors:** Sivesh K. Kamarajah, Anantha Madhavan, Jakub Chmelo, Maziar Navidi, Shajahan Wahed, Arul Immanuel, Nick Hayes, S. Michael Griffin, Alexander W. Phillips

**Affiliations:** 1 Northern Oesophagogastric Unit, Royal Victoria Infirmary, Newcastle University Trust Hospitals, Newcastle-Upon-Tyne, UK; 2 Institute of Cellular Medicine, Newcastle University, Newcastle-Upon-Tyne, UK; 3 School of Medical Education, Newcastle University, Newcastle-Upon-Tyne, UK

## Abstract

**Introduction:**

Esophagectomy is a key component in the curative treatment of esophageal cancer. Little is understood about the impact of smoking status on perioperative morbidity and mortality and the long-term outcome of patients following esophagectomy.

**Objective:**

This study aimed to evaluate morbidity and mortality according to smoking status in patients undergoing esophagectomy for esophageal cancer.

**Methods:**

Consecutive patients undergoing two-stage transthoracic esophagectomy (TTE) for esophageal cancers (adenocarcinoma or squamous cell carcinoma) between January 1997 and December 2016 at the Northern Oesophagogastric Unit were included from a prospectively maintained database. The main explanatory variable was smoking status, defined as current smoker, ex-smoker, and non-smoker. The primary outcome was overall survival (OS), while secondary outcomes included perioperative complications (overall, anastomotic leaks, and pulmonary complications) and survival (cancer-specific survival [CSS], recurrence-free survival [RFS]).

**Results:**

During the study period, 1168 patients underwent esophagectomy for cancer. Of these, 24% (*n* = 282) were current smokers and only 30% (*n* = 356) had never smoked. The median OS of current smokers was significantly shorter than ex-smokers and non-smokers (median 36 vs. 42 vs. 48 months; *p* = 0.015). However, on adjusted analysis, there was no significant difference in long-term OS between smoking status in the entire cohort. The overall complication rates were significantly higher with current smokers compared with ex-smokers or non-smokers (73% vs. 66% vs. 62%; *p* = 0.018), and there were no significant differences in anastomotic leaks and pulmonary complications between the groups. On subgroup analysis by receipt of neoadjuvant therapy and tumor histology, smoking status did not impact long-term survival in adjusted multivariable analyses.

**Conclusion:**

Although smoking is associated with higher rates of short-term perioperative morbidity, it does not affect long-term OS, CSS, and RFS following esophagectomy for esophageal cancer. Therefore, implementation of perioperative pathways to optimize patients may help reduce the risk of complications.

**Supplementary Information:**

The online version contains supplementary material available at 10.1245/s10434-021-09720-6.

Esophagectomy remains a key part of the treatment for patients with potentially curable esophageal cancer; however, esophagectomy is a technically demanding procedure and is associated with a high incidence of postoperative morbidity and mortality.^1^–^5^ Anastomotic leaks and pulmonary complications remain a major cause of postoperative mortality.^6^–^9^ Thus, identifying those at risk of pulmonary morbidities and optimizing their perioperative management has an important role for patients being considered for esophagectomy.

Because smoking is recognized as a risk factor for morbidity following esophagectomy, particularly with respect to pulmonary complications,^7^^,^^9^–^12^ smoking cessation has been demonstrated to reduce postoperative pulmonary morbidity.^13^^,^^14^ However, the impact of smoking status on long-term survival is unclear and is limited to small case series from eastern cohorts focused on esophageal squamous cell carcinoma (SCC).^5^^,^^15^ A recent analysis of 5354 esophagectomies from a Japanese nationwide web-based database demonstrated that smoking within 1 year of undergoing esophagectomy is an independent risk factor for 30-day mortality.^5^

Despite this, its impact on long-term survival has not been fully established. The aim of this study was to investigate the impact of smoking status on long-term survival in patients undergoing esophagectomy. These findings will be useful for counseling patients and targeting prehabilitation in patients undergoing thoracoabdominal surgery.

## Methods

### Study Population

Consecutive patients undergoing two-stage transthoracic esophagectomy (TTE) for esophageal cancer (adenocarcinoma or SCC) were included in this study. Patients were identified from the Northern Oesophagogastric Unit (NOGU) in Newcastle-upon-Tyne between January 1997 and December 2016 through a contemporaneously maintained database. Patients with metastases, non-resectable tumors during exploratory surgery, or macroscopically incomplete resections (R2) were excluded.

### Staging

Patients were staged using esophagogastroduodenoscopy (OGD) with biopsy, endoscopic ultrasonography, and thoracoabdominal computerized tomography (CT). Positron emission tomography (PET) CT scan became routine for patients during the study period. Laparoscopy with peritoneal washings was used for junctional tumors. All cases were discussed by the multidisciplinary team and treatment recommendations were discussed with patients. All patients in this study were staged according to the American Joint Committee on Cancer (AJCC) 8th edition.^16^

### Neoadjuvant Therapy and Operative Approach

Multiple neoadjuvant regimens were employed in the present study, determined by the standard of care as practiced in the UK, and recruiting trials at the time of each patient’s treatment. The most commonly used regimen was a combination of epirubicin, cisplatin, and capecitabine (ECX), over the course of this study.^17^^,^^18^ TTE with two- or three-field lymphadenectomy was carried out 4–6 weeks after completion of neoadjuvant therapy using a conventional approach as previously reported.^19^ As per protocol, after surgery, each lymph node station was dissected from the specimen by the operating surgeon and placed into individual pots for analysis by the pathologist.^20^

### Pathology

Histopathological reporting was carried out by specialist gastrointestinal pathologists using a standardized proforma in line with guidelines produced by the Royal College of Pathologists;^21^ this included tumor type and differentiation, depth of tumor infiltration, and degree of tumor regression according to the Mandard criteria. The total number of lymph nodes and number of nodal metastases from each location was also recorded.

### Follow-Up and Definition of Recurrence

Smoking status was defined as follows: (1) current smokers were defined as ongoing smokers at the time of diagnosis, or had stopped within 6 weeks of surgery; (2) ex-smokers were those who stopped >6 weeks prior to surgery; and (3) non-smokers were defined as those who have never smoked, consistent with previously published multicenter studies.^22^–^24^ Patients were reviewed in the outpatient clinic at 3- to 6-month intervals during the first 2 years and every 6 months or annually for 5 years, then yearly until 10 years post initial surgery. Recurrence of disease suspected on clinical grounds was confirmed with either CT or endoscopy.

### Statistical Analysis

Categorical variables were compared using the Kruskal–Wallis test, and non-normally distributed data were analyzed using the Mann–Whitney U test. Survival was estimated using Kaplan–Meier survival curves and compared using the log-rank test. A *p*-value <0.05 was considered statistically significant. Data analysis was performed using R Foundation Statistical software (R 3.2.2) with TableOne, ggplot2, Hmisc, and survival packages (The R Foundation for Statistical Computing, Vienna, Austria) as previously described.^19^^,^^25^^,^^26^

## Results

### Baseline Characteristics

During the study period, 1168 patients underwent esophagectomy for cancer, of whom 282 were current (24%) smokers and 530 (45%) were ex-smokers. Current smokers were more likely to be younger (median 62 vs. 67 vs. 67 years; *p* < 0.001), have an SCC (27% vs. 19% vs. 28%; *p* = 0.005), be from more deprived groups (Index of Multiple Deprivation [IMD] 1/2: 40% vs. 39% vs. 42%; *p* < 0.001), and have higher American Society of Anesthesiologists (ASA) grade 3/4 (34% vs. 26% vs. 21%; *p* = 0.003) than ex-smokers or never smokers. There were no significant differences in overall pathological stage, tumor grades, lymphadenectomy, and margin-positive rates between groups. Clinicopathologic variables are presented in Table 1. Trends in the rates of two-stage TTE and neoadjuvant therapy are presented in electronic supplementary Fig. 1. The two-stage TTE rates over the past 2 decades ranged from 92% to 100%.

**Table 1:** Baseline demographics and postoperative outcomes of all patients undergoing transthoracic esophagectomy for esophageal cancer, stratified by smoking status

		Current [*n* = 282]	Ex-smoker [*n* = 530]	Never [*n* = 356]	Total [*n* = 1168]	*p* value
Age at diagnosis, years	Median (IQR)	62.0 (14.0)	66.5 (11.0)	67.0 (13.2)	65.0 (13.0)	< 0.001
Sex	Male	204 (72.3)	430 (81.1)	228 (64.0)	862 (73.8)	< 0.001
	Female	78 (27.7)	100 (18.9)	128 (36.0)	306 (26.2)	
Histology	Adenocarcinoma	205 (72.7)	428 (80.8)	258 (72.5)	891 (76.3)	0.005
	SCC	77 (27.3)	102 (19.2)	98 (27.5)	277 (23.7)	
Body mass index, kg/m^2^	Median (IQR)	24.2 (6.9)	26.8 (5.6)	26.0 (5.6)	26.0 (6.0)	< 0.001
IMD decile	1	86 (30.5)	122 (23.0)	51 (14.3)	259 (22.2)	< 0.001
	2	26 (9.2)	84 (15.8)	98 (27.5)	208 (17.8)	
	3	77 (27.3)	113 (21.3)	75 (21.1)	265 (22.7)	
	4	45 (16.0)	110 (20.8)	65 (18.3)	220 (18.8)	
	5	35 (12.4)	80 (15.1)	59 (16.6)	174 (14.9)	
	Unknown	13 (4.6)	21 (4.0)	8 (2.2)	42 (3.6)	
ASA grade	1	33 (11.7)	70 (13.2)	72 (20.2)	175 (15.0)	0.003
	2	125 (44.3)	276 (52.1)	180 (50.6)	581 (49.7)	
	3	92 (32.6)	134 (25.3)	70 (19.7)	296 (25.3)	
	4	3 (1.1)	3 (0.6)	2 (0.6)	8 (0.7)	
	Unknown	29 (10.3)	47 (8.9)	32 (9.0)	108 (9.2)	
Overall treatment	NAC + surgery	145 (51.4)	282 (53.2)	174 (48.9)	601 (51.5)	0.449
	Surgery only	137 (48.6)	248 (46.8)	182 (51.1)	567 (48.5)	
AJCC pathological stage classification	0	16 (5.7)	34 (6.4)	23 (6.5)	73 (6.2)	0.304
	I	53 (18.8)	114 (21.5)	81 (22.8)	248 (21.2)	
	II	49 (17.4)	118 (22.3)	86 (24.2)	253 (21.7)	
	III	134 (47.5)	219 (41.3)	139 (39.0)	492 (42.1)	
	IVA	30 (10.6)	45 (8.5)	27 (7.6)	102 (8.7)	
Tumor grade	Well	26 (9.2)	39 (7.4)	36 (10.1)	101 (8.6)	0.216
	Moderate	127 (45.0)	264 (49.8)	165 (46.3)	556 (47.6)	
	Poor	111 (39.4)	187 (35.3)	118 (33.1)	416 (35.6)	
	Unknown	18 (6.4)	40 (7.5)	37 (10.4)	95 (8.1)	
Lymph nodes examined	Median (IQR)	31.0 (17.0)	29.0 (15.5)	30.0 (13.0)	30.0 (15.2)	0.260
Margin status	R0	272 (96.5)	524 (98.9)	347 (97.5)	1143 (97.9)	0.064
	R1	10 (3.5)	6 (1.1)	9 (2.5)	25 (2.1)	
Lymphatic involvement	No	149 (52.8)	289 (54.5)	203 (57.0)	641 (54.9)	0.559
	Yes	133 (47.2)	241 (45.5)	153 (43.0)	527 (45.1)	
Venous involvement	No	181 (64.2)	337 (63.6)	246 (69.1)	764 (65.4)	0.211
	Yes	101 (35.8)	193 (36.4)	110 (30.9)	404 (34.6)	
Perineural involvement	No	144 (51.1)	296 (55.8)	212 (59.6)	652 (55.8)	0.100
	Yes	138 (48.9)	234 (44.2)	144 (40.4)	516 (44.2)	
Extracapsular spread	No	242 (85.8)	445 (84.0)	296 (83.1)	983 (84.2)	0.647
	Yes	40 (14.2)	85 (16.0)	60 (16.9)	185 (15.8)	
Critical care stay	Median (IQR)	3.0 (7.0)	2.0 (4.0)	2.0 (3.0)	2.0 (4.0)	< 0.001
Length of stay	Median (IQR)	15.0 (11.0)	15.0 (10.8)	15.0 (9.0)	15.0 (10.0)	0.515
Overall complications	No	76 (27.0)	181 (34.2)	136 (38.2)	393 (33.6)	0.011
	Yes	206 (73.0)	349 (65.8)	220 (61.8)	775 (66.4)	
Surgical site infection	No	251 (89.0)	477 (90.0)	325 (91.3)	1053 (90.2)	0.621
	Yes	31 (11.0)	53 (10.0)	31 (8.7)	115 (9.8)	
Pulmonary complications	No	244 (86.5)	471 (88.9)	321 (90.2)	1036 (88.7)	0.348
	Yes	38 (13.5)	59 (11.1)	35 (9.8)	132 (11.3)	
Cardiac complications	No	269 (95.4)	494 (93.2)	328 (92.1)	1091 (93.4)	0.250
	Yes	13 (4.6)	36 (6.8)	28 (7.9)	77 (6.6)	
Anastomotic leaks	No	253 (89.7)	483 (91.1)	334 (93.8)	1070 (91.6)	0.154
	Yes	29 (10.3)	47 (8.9)	22 (6.2)	98 (8.4)	
In-hospital mortality	No	267 (94.7)	516 (97.4)	342 (96.1)	1125 (96.3)	0.149
	Yes	15 (5.3)	14 (2.6)	14 (3.9)	43 (3.7)	
30-day mortality	No	273 (96.8)	520 (98.1)	344 (96.6)	1137 (97.3)	0.328
	Yes	9 (3.2)	10 (1.9)	12 (3.4)	31 (2.7)	

Data are expressed as *n* (%) unless otherwise specified

*IMD* Index of Multiple Deprivation, *ASA* American Society of Anesthesiologists, *AJCC* American Joint Committee on Cancer, *IQR* interquartile range, *SCC* squamous cell carcinoma, *NAC* neoadjuvant chemotherapy

### Postoperative Outcomes and Survival

**Fig. 1:**
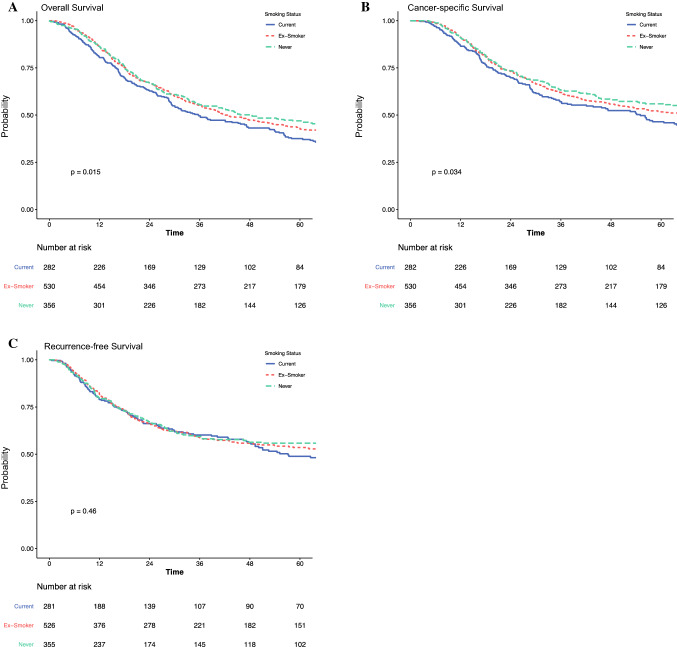
Impact of smoking on (**a**) overall survival, (**b**) cancer-specific survival, and (**c**) recurrence-free survival, in patients undergoing transthoracic esophagectomy for esophageal cancer

Trends in the rates of overall complications and anastomotic leaks are presented in electronic supplementary Fig. 1. Current smokers had significantly higher rates of overall complications compared with ex-smokers and never smokers (73% vs. 66% vs. 62%, *p* = 0.011); however, there were no significant differences in the rates of surgical site infections, pulmonary complications, cardiac complications, or anastomotic leaks between the groups. The median overall survival (OS; median 36 vs. 42 vs. 48 months, *p* = 0.015) (Fig. 1a) and cancer-specific survival (CSS; median 55 vs. 68 vs. 119 months; *p* = 0.034) (Fig. 1b) of current smokers was significantly shorter than ex-smokers and non-smokers. There were no statistical differences on recurrence-free survival (RFS) between smoking status groups (Fig. 1c). On adjusted Cox regression, smoking statuses were not independent prognostic factors for OS, CSS, and RFS (Table 2, electronic supplementary Tables 1–3).

**Table 2:** Impact of smoking on long-term overall, cancer-specific, and recurrence-free survival following transthoracic esophagectomy for esophageal cancers

	No. of patients	Median survival, months	Univariable HR (95% CI)	Multivariable HR (95% CI)
*Overall survival*				
Current	282	35.6 (29.3–47.3)	REF	REF
Ex-smoker	530	42.0 (37.0–53.3)	0.85 (0.71–1.01), *p* = 0.060	0.94 (0.77–1.14), *p* = 0.499
Never	356	48.3 (36.9–76.9)	0.76 (0.62–0.92), *p* = 0.004	0.93 (0.75–1.16), *p* = 0.529
*Cancer-specific survival*				
Current	282	55.0 (37.6–68.8)	REF	REF
Ex-smoker	530	68.2 (52.3–170.5)	0.83 (0.67–1.01), *p* = 0.068	0.94 (0.75–1.19), *p* = 0.615
Never	356	118.6 (62.2–NR)	0.74 (0.59–0.93), *p* = 0.011	0.90 (0.70–1.16), *p* = 0.420
*Recurrence-free survival*				
Current	281	57.1 (47.7–NR)	REF	REF
Ex-smoker	526	NR (54.2–NR)	0.90 (0.72–1.13), *p* = 0.377	1.00 (0.78–1.29), *p* = 0.980
Never	355	NR (76.0–NR)	0.84 (0.65–1.08), *p* = 0.173	1.03 (0.78–1.36), *p* = 0.842

*HR* hazard ratio, *CI* confidence interval, *NR* not reached

### Interaction Between Smoking Status and Neoadjuvant Therapy

Of the entire cohort, 601 patients received neoadjuvant therapy. Interaction analyses were performed to further understand the impact of smoking status in patients receiving neoadjuvant therapy. Baseline demographics and postoperative outcomes of patients with and without neoadjuvant therapy are presented in electronic supplementary Tables 4 and 5, respectively. As above, similar trends were observed for age, IMD decile, and ASA grade between the different smoking status groups in patients with neoadjuvant therapy. The overall complication rates were significantly higher in current smokers compared with ex-smokers and never smokers in patients receiving one therapy (72% vs. 64% vs. 56%; *p* = 0.012) [electronic supplementary Table 4), but this was not the case for patients without neoadjuvant therapy (74% vs. 69% vs. 67%, *p* = 0.4).

**Fig. 2:**
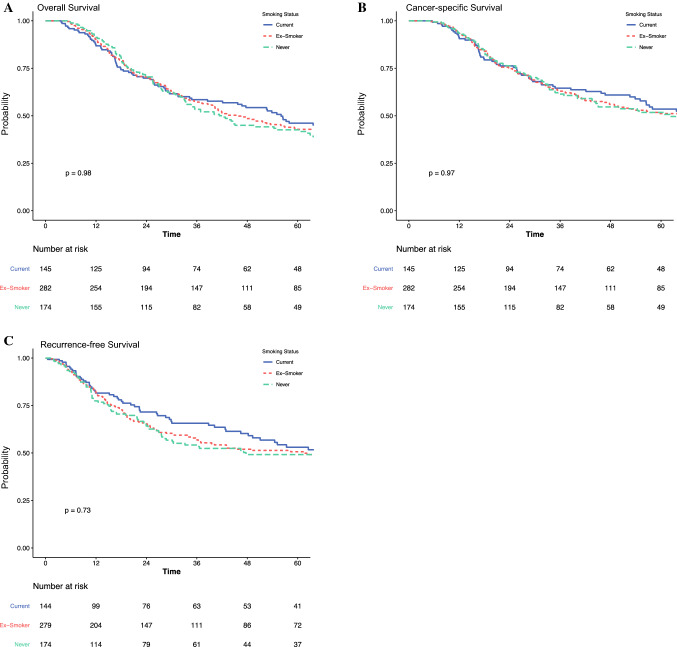
Impact of smoking on (**a**) overall survival, (**b**) cancer-specific survival, and (**c**) recurrence-free survival, in patients undergoing neoadjuvant therapy and transthoracic esophagectomy for esophageal cancer

**Fig. 3:**
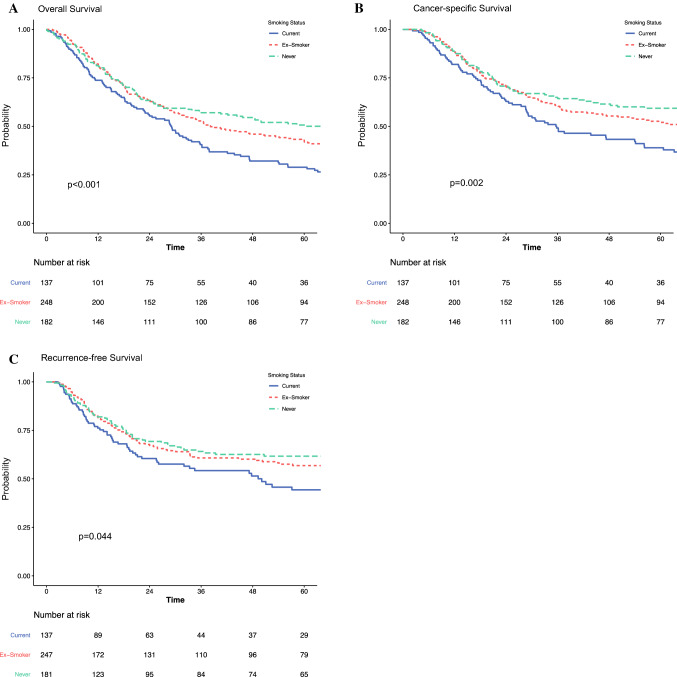
Impact of smoking on (**a**) overall survival, (**b**) cancer-specific survival, and (**c**) recurrence-free survival, in patients undergoing transthoracic esophagectomy only for esophageal cancer

In patients with neoadjuvant therapy, there were no significant differences in OS for current smokers compared with ex-smokers and never smokers (median 56 vs. 46 vs. 42; *p* = 1.0) (Table 3, Fig. 2a). There were also no significant differences for CSS (Table 3, Fig. 2b) and RFS (Table 3, Fig. 2c). These results were consistent on multivariable analysis, as presented in Table 3. In contrast, current smokers undergoing unimodality surgery only had significantly shorter survival than ex-smokers or never smokers, for OS (median 29 vs. 38 vs. 70 months; *p* < 0.001) (Table 3, Fig. 3a), CSS (median 36 vs. 78 vs. 200 months; *p* = 0.002) (Table 3, Fig. 3b), and RFS (median 50 vs. not reached vs. not reached; *p* = 0.044) (Table 3, Fig. 3c). These results were consistent on multivariable analysis, as presented in Table 3. In multivariable analyses modeling the interaction between receipt of neoadjuvant therapy and smoking status, there were no survival differences in ex-smokers or never smokers, by neoadjuvant therapy status, for OS, CSS, and RFS.

**Table 3:** Subset analyses, by neoadjuvant therapy, on the impact of smoking on long-term overall, cancer-specific, and recurrence-free survival following transthoracic esophagectomy for esophageal cancers

	No. of patients	Median survival, months	Univariable HR (95% CI)	Multivariable HR (95% CI)
**Neoadjuvant and surgery**				
*Overall survival*				
Current	145	56.0 (38.5–67.9)	REF	REF
Ex-smoker	282	46.0 (39.8–59.2)	1.02 (0.79–1.32), *p* = 0.877	1.10 (0.82–1.48), *p* = 0.513
Never	174	42.2 (33.4–62.2)	1.00 (0.75–1.33), *p* = 0.999	1.09 (0.80–1.51), *p* = 0.582
*Cancer-specific survival*				
Current	145	69.7 (55.0–NR)	REF	REF
Ex-smoker	282	67.5 (48.9–NR)	1.03 (0.77–1.39), *p* = 0.822	1.17 (0.83–1.65), *p* = 0.372
Never	174	62.2 (44.2–NR)	1.01 (0.73–1.40), *p* = 0.957	1.07 (0.74–1.55), *p* = 0.720
*Recurrence-free survival*				
Current	144	81.4 (49.3–NR)	REF	REF
Ex-smoker	279	61.5 (36.4–NR)	1.13 (0.82–1.55), *p* = 0.451	1.19 (0.83–1.69), *p* = 0.346
Never	174	47.7 (28.2–NR)	1.12 (0.79–1.59), *p* = 0.524	1.20 (0.82–1.76), *p* = 0.351
**Unimodality surgery only**				
*Overall survival*				
Current	137	29.1 (22.8–35.8)	REF	REF
Ex-smoker	248	38.2 (31.5–59.4)	0.72 (0.57–0.91), *p* = 0.006	0.86 (0.66–1.13), *p* = 0.278
Never	182	69.5 (40.5–111.3)	0.61 (0.47–0.78), *p* < 0.001	0.86 (0.63–1.18), *p* = 0.359
*Cancer-specific survival*				
Current	137	35.8 (28.7–56.2)	REF	REF
Ex-smoker	248	78.2 (46.5–186.0)	0.68 (0.51–0.91), *p* = 0.009	0.81 (0.58–1.14), *p* = 0.229
Never	182	199.8 (88.1–NR)	0.57 (0.42–0.79), *p* = 0.001	0.79 (0.53–1.17), *p* = 0.236
*Recurrence-free survival*				
Current	137	50.1 (26.1–NR)	REF	REF
Ex-smoker	247	NR (NR–NR)	0.72 (0.52–1.00), *p* = 0.052	0.89 (0.61–1.31), *p* = 0.560
Never	181	NR (NR–NR)	0.65 (0.45–0.93), *p* = 0.019	1.02 (0.66–1.59), *p* = 0.918

*HR* hazard ratio, *CI* confidence interval, *NR* not reached

### Subgroup Analysis by Tumor Histology

Interaction analyses were performed to further understand the impact of smoking status by tumor histology (adenocarcinoma and SCC). Baseline demographics and postoperative outcomes for esophageal adenocarcinoma and SCC are presented in electronic supplementary Tables 6 and 7, respectively. As above, similar trends were observed for age and IMD decile between the different smoking status groups in patients with adenocarcinoma and SCC.

In patients with esophageal adenocarcinoma, there were no significant differences in OS for current smokers compared with ex-smokers and never smokers (median 38 vs. 42 vs. 40; *p* = 0.4) (Table 4, electronic supplementary Fig. 2a). There were also no significant differences for CSS (Table 4, electronic supplementary Fig. 2b) and RFS (Table 4, electronic supplementary Fig. 2c). These results were consistent with adjusted Cox multivariable regression analysis, as presented in Table 4. In contrast, for esophageal SCC, current smokers had significantly shorter survival than ex-smokers or never smokers, for OS (median 34 vs. 52 vs. 96 months; *p* = 0.001) (Table 4, electronic supplementary Fig. 3a) and CSS (median 47 vs. 68 vs. 154 months; *p* = 0.040) (Table 4, electronic supplementary Fig. 3b), but not RFS (Table 3, electronic supplementary Fig. 3c). On adjusted Cox regression analyses, there was no significant impact of smoking on OS, CSS, and RFS, as presented in Table 4. In multivariable analyses modeling the interaction between receipt of neoadjuvant therapy and smoking status, there were no survival differences in ex-smokers or never smokers, by neoadjuvant therapy status, for OS, CSS, and RFS.

**Table 4:** Subset analyses, by tumor histology, on the impact of smoking on long-term overall, cancer-specific, and recurrence-free survival following transthoracic esophagectomy for esophageal cancers

	No. of patients	Median survival, months	Univariable HR (95% CI)	Multivariable HR (95% CI)
**Adenocarcinoma**				
*Overall survival*				
Current	205	37.9 (28.9–56.0)	REF	REF
Ex-smoker	428	42.0 (36.9–52.6)	0.89 (0.73–1.09), *p* = 0.260	0.94 (0.75–1.18), *p* = 0.585
Never	258	40.1 (33–56.2)	0.87 (0.70–1.09), *p* = 0.232	1.00 (0.78–1.29), *p* = 1.000
*Cancer-specific survival*				
Current	205	55.0 (38.5–81.5)	REF	REF
Ex-smoker	428	68.5 (49.7–170.5)	0.83 (0.65–1.05), *p* = 0.115	0.86 (0.66–1.12), *p* = 0.272
Never	258	86.8 (45.1–NR)	0.81 (0.62–1.05), *p* = 0.110	0.86 (0.64–1.16), *p* = 0.315
*Recurrence-free survival*				
Current	204	51.1 (42.7–NR)	REF	REF
Ex-smoker	424	NR (49.0–NR)	0.89 (0.69–1.15), *p* = 0.373	0.91 (0.69–1.22), *p* = 0.540
Never	258	NR (36.6–NR)	0.88 (0.66–1.18), *p* = 0.401	0.97 (0.70–1.33), *p* = 0.844
**Squamous cell carcinoma**				
*Overall survival*				
Current	77	33.8 (28.6–47.2)	REF	REF
Ex-smoker	102	51.6 (28.8–85.7)	0.73 (0.51–1.04), *p* = 0.082	0.91 (0.60–1.38), *p* = 0.648
Never	98	96.2 (58.3–NR)	0.48 (0.32–0.70), *p* < 0.001	0.77 (0.48–1.24), *p* = 0.278
*Cancer-specific survival*				
Current	77	46.7 (33.8–NR)	REF	REF
Ex-smoker	102	68.2 (40.9–NR)	0.81 (0.53–1.25), *p* = 0.342	1.10 (0.65–1.86), *p* = 0.711
Never	98	153.6 (96.2–NR)	0.55 (0.35–0.88), *p* = 0.013	1.09 (0.60–1.98), *p* = 0.780
*Recurrence-free survival*				
Current	77	97.2 (48.2–NR)	REF	REF
Ex-smoker	102	NR (37.0–NR)	0.91 (0.56–1.49), *p* = 0.716	1.04 (0.59–1.86), *p* = 0.882
Never	97	NR (NR–NR)	0.73 (0.43–1.22), *p* = 0.224	1.42 (0.75–2.68), *p* = 0.280

*HR* hazard ratio, *CI* confidence interval, *NR* not reached

## Discussion

This single-center study analysis from a high-volume unit in patients undergoing TTE demonstrates that ongoing smoking significantly reduces long-term survival in patients with an SCC and also in those who receive unimodality surgery. However, a higher number of complications were seen in patients who received neoadjuvant treatment, although this does not translate to higher rates of anastomotic leaks or pulmonary complications, which might be expected, and likely reflects the overall deconditioning associated with neoadjuvant therapy.^27^ These findings are relevant in counseling patients preoperatively on their expected operative and oncological outcomes following surgery. Furthermore, interventions to reduce smoking are needed and prehabilitation pathways for these high-risk groups may improve outcomes in smokers and ex-smokers, as both groups have similar operative and oncological profiles compared with non-smokers.^28^

Although smoking is a well-established risk factor for esophageal SCC and adenocarcinoma,^29^ little is known about the impact of smoking on long-term survival. Until recently, most studies have focused on the relationship between smoking and cancer incidence.^30^–^32^ A nationwide case-control study in Sweden first reported that ex-smokers had a worse outcome for esophageal SCC, but a current smoker status was not statistically significant (hazard ratio 1.4, 95% confidence interval 0.7–2.8).^33^ In contrast, Sun et al.^15^ (*n* = 488 patients) demonstrated that smokers were associated with poor long-term survival, but not disease-free survival, following esophagectomy for esophageal SCC. In the present study, the survival of current smokers was significantly shorter than ex-smokers and non-smokers (median 35 vs. 43 vs. 48 months; *p* = 0.016); however, on adjusted analysis, there was no significant difference in long-term survival between smoking status in the entire cohort.

It is difficult to fully explain why patients with an SCC appeared to be more compromised by being current smokers than those with an adenocarcinoma. Underlying mechanisms on the impact of smoking on long-term survival is not well understood. Several mechanisms have been investigated to explain these findings. Recent data from the field of epigenetics show that active smoking is associated with an aberrant DNA methylation pattern, which is linked to carcinogenesis and poor oncological outcomes.^34^–^36^ Shui et al. demonstrated that aberrant DNA methylation in key gene promoters associated with active smoking can lead to tumor recurrence in patients with prostate cancer.^34^ Their study also suggested that this process might be reversible following smoking cessation.^34^ The most severe impact of smoking was found in patients treated with chemotherapy, which might be explained by the reduced chemosensitivity of esophageal cancer cells exposed to nicotine, previously described in *in vitro* research reports.^37^^,^^38^ However, in our study, there were no significant differences in survival between smokers and never smokers receiving neoadjuvant therapy even when stratified by tumor response, highlighting variability in the genetic landscape of these tumors.

This study has a number of limitations to address. First, the data on pack-year history could have been more comprehensive. It may be that a certain threshold exists where patients are significantly affected. Furthermore, it would be good to establish whether a specific time frame exists for ex-smokers where a benefit develops. Second, it was impossible to capture whether all patients had any advice or intervention for smoking cessation and the compliance rates during neoadjuvant therapy and/or surgery. Third, this study was not able to incorporate better definitions of smoking, such as the Brinkman index, to allow refined analysis. Finally, we were unable to capture whether recurrent smoking in patients who stopped smoking >6 weeks prior to surgery had an impact on long-term survival. Nevertheless, this study represents a large study evaluating the long-term impact of smoking on OS.

## Conclusion

This study demonstrates that for patients with adenocarcinoma undergoing unimodality surgery, and for all SCC patients, current smokers have significantly poorer long-term survival compared with ex-smokers or non-smokers. However, there is no survival difference in patients who receive neoadjuvant therapy for adenocarcinoma, which is contrary to in vitro reports. This warrants additional investigation to further delineate the genetic landscape of esophageal cancers to identify high-risk groups that may warrant further multimodality therapy.

## Supplementary Information

Below is the link to the electronic supplementary material.

Supplementary file1 (DOCX 1189 kb)
