# Supplementary material for: Impact of Smoking Status on Perioperative Morbidity, Mortality, and Long-Term Survival Following Transthoracic Esophagectomy for Esophageal Cancer
Source: Ann Surg Oncol. 2021 Mar 3;28(9):4905–15. doi: 10.1245/s10434-021-09720-6 (PMC8349321; doi:10.1245/s10434-021-09720-6)
Supplement: Supplementary file 1 — Supplementary file1 (DOCX 1189 kb) [file 10434_2021_9720_MOESM1_ESM.docx]

Supplementary Table 1 Cox regression analyses on smoking status on overall survival after transthoracic esophagectomy for esophageal cancer

|  |  | HR (univariable) | HR (multivariable) |
| --- | --- | --- | --- |
| Smoking Status | Current | - | - |
|  | Ex-Smoker | 0.85 (0.71-1.01, p=0.060) | 0.94 (0.77-1.14, p=0.499) |
|  | Never | 0.76 (0.62-0.92, p=0.004) | 0.93 (0.75-1.16, p=0.529) |
| Age at Diagnosis | Mean (SD) | 1.02 (1.01-1.03, p<0.001) | 1.01 (1.00-1.02, p=0.028) |
| Gender | Male | - | - |
|  | Female | 0.79 (0.66-0.93, p=0.004) | 0.67 (0.55-0.82, p<0.001) |
| Histology | Adenocarcinoma | - | - |
|  | SCC | 0.97 (0.82-1.15, p=0.746) | 1.28 (1.03-1.59, p=0.025) |
| Body Mass Index, kg/m2 | Mean (SD) | 0.96 (0.95-0.98, p<0.001) | 0.97 (0.96-0.99, p=0.003) |
| IMD Decile | 1 | - | - |
|  | 2 | 0.90 (0.72-1.13, p=0.381) | 0.95 (0.74-1.22, p=0.688) |
|  | 3 | 0.97 (0.79-1.20, p=0.804) | 0.95 (0.77-1.19, p=0.675) |
|  | 4 | 0.82 (0.65-1.02, p=0.076) | 0.98 (0.77-1.24, p=0.843) |
|  | 5 | 0.72 (0.56-0.92, p=0.008) | 0.74 (0.57-0.96, p=0.023) |
|  | Unknown | 2.13 (1.47-3.09, p<0.001) | 2.49 (1.66-3.75, p<0.001) |
| ASA Grade | Grade 1 | - | - |
|  | Grade 2 | 1.13 (0.91-1.40, p=0.268) | 1.18 (0.94-1.49, p=0.151) |
|  | Grade 3 | 1.44 (1.14-1.81, p=0.002) | 1.26 (0.97-1.63, p=0.078) |
|  | Grade 4 | 1.59 (0.70-3.62, p=0.268) | 1.57 (0.68-3.64, p=0.293) |
|  | Unknown | 1.27 (0.95-1.70, p=0.100) | 1.13 (0.81-1.56, p=0.474) |
| Overall Treatment | NAC + Surgery | - | - |
|  | Surgery Only | 1.11 (0.96-1.29, p=0.148) | 1.35 (1.13-1.60, p=0.001) |
| AJCC Pathological Stage Classification | Stage 0 | - | - |
|  | Stage I | 1.07 (0.68-1.67, p=0.778) | 1.48 (0.81-2.70, p=0.199) |
|  | Stage II | 1.33 (0.85-2.08, p=0.211) | 1.44 (0.79-2.61, p=0.230) |
|  | Stage III | 4.10 (2.69-6.24, p<0.001) | 3.46 (1.92-6.23, p<0.001) |
|  | Stage IVA | 6.99 (4.42-11.05, p<0.001) | 4.49 (2.39-8.46, p<0.001) |
| Tumor Grade | Well | - | - |
|  | Moderate | 1.75 (1.31-2.33, p<0.001) | 1.24 (0.90-1.70, p=0.193) |
|  | Poor | 2.69 (2.01-3.61, p<0.001) | 1.51 (1.08-2.10, p=0.016) |
|  | Unknown | 1.37 (0.93-2.02, p=0.109) | 1.43 (0.86-2.37, p=0.163) |
| Lymph Nodes Examined | Mean (SD) | 1.00 (0.99-1.00, p=0.472) | 0.99 (0.98-1.00, p=0.022) |
| Margin Status | R0 | - | - |
|  | R1 | 4.65 (3.08-7.03, p<0.001) | 2.13 (1.30-3.49, p=0.003) |
| Lymphatic Involvement | No | - | - |
|  | Yes | 2.36 (2.04-2.73, p<0.001) | 1.36 (1.12-1.65, p=0.002) |
| Venous Involvement | No | - | - |
|  | Yes | 2.08 (1.80-2.41, p<0.001) | 0.92 (0.76-1.11, p=0.402) |
| Perineural Involvement | No | - | - |
|  | Yes | 2.64 (2.28-3.05, p<0.001) | 1.51 (1.25-1.83, p<0.001) |
| Extracapsular Spread | No | - | - |
|  | Yes | 2.54 (2.13-3.04, p<0.001) | 1.74 (1.40-2.17, p<0.001) |

Supplementary Table 2 Cox regression analyses on smoking status on cancer-specific survival after esophagectomy for esophageal cancer

|  |  | HR (univariable) | HR (multivariable) |
| --- | --- | --- | --- |
| Smoking Status | Current | - | - |
|  | Ex-Smoker | 0.83 (0.67-1.01, p=0.068) | 0.94 (0.75-1.19, p=0.615) |
|  | Never | 0.74 (0.59-0.93, p=0.011) | 0.90 (0.70-1.16, p=0.420) |
| Age at Diagnosis | Mean (SD) | 1.01 (1.00-1.01, p=0.287) | 1.00 (0.99-1.01, p=0.565) |
| Gender | Male | - | - |
|  | Female | 0.78 (0.64-0.95, p=0.013) | 0.70 (0.55-0.89, p=0.003) |
| Histology | Adenocarcinoma | - | - |
|  | SCC | 0.93 (0.76-1.14, p=0.463) | 1.34 (1.03-1.74, p=0.028) |
| Body Mass Index, kg/m2 | Mean (SD) | 0.97 (0.95-0.99, p=0.002) | 0.99 (0.97-1.01, p=0.570) |
| IMD Decile | 1 | - | - |
|  | 5 | 1.09 (0.84-1.42, p=0.512) | 1.15 (0.86-1.55, p=0.350) |
|  | 2 | 1.11 (0.86-1.42, p=0.426) | 1.10 (0.84-1.43, p=0.485) |
|  | 3 | 0.88 (0.67-1.15, p=0.343) | 1.05 (0.78-1.42, p=0.733) |
|  | 4 | 0.77 (0.57-1.04, p=0.092) | 0.76 (0.55-1.06, p=0.103) |
|  | Unknown | 2.50 (1.63-3.82, p<0.001) | 2.73 (1.70-4.39, p<0.001) |
| ASA Grade | Grade 1 | - | - |
|  | Grade 2 | 0.98 (0.77-1.25, p=0.887) | 1.02 (0.78-1.33, p=0.886) |
|  | Grade 3 | 1.12 (0.86-1.47, p=0.390) | 0.93 (0.69-1.26, p=0.653) |
|  | Grade 4 | 0.61 (0.15-2.48, p=0.492) | 0.52 (0.12-2.13, p=0.360) |
|  | Unknown | 1.19 (0.86-1.67, p=0.297) | 1.04 (0.71-1.53, p=0.840) |
| Overall Treatment | NAC + Surgery | - | - |
|  | Surgery Only | 1.05 (0.89-1.25, p=0.546) | 1.41 (1.15-1.73, p=0.001) |
| AJCC Pathological Stage Classification | Stage 0 | - | - |
|  | Stage I | 1.02 (0.53-1.96, p=0.955) | 1.69 (0.67-4.30, p=0.269) |
|  | Stage II | 1.68 (0.89-3.17, p=0.108) | 2.12 (0.85-5.31, p=0.108) |
|  | Stage III | 6.44 (3.53-11.75, p<0.001) | 6.40 (2.58-15.86, p<0.001) |
|  | Stage IVA | 11.62 (6.18-21.86, p<0.001) | 8.75 (3.42-22.38, p<0.001) |
| Tumor Grade | Well | - | - |
|  | Moderate | 2.33 (1.56-3.47, p<0.001) | 1.37 (0.88-2.13, p=0.166) |
|  | Poor | 3.73 (2.50-5.57, p<0.001) | 1.69 (1.07-2.67, p=0.023) |
|  | Unknown | 1.66 (0.99-2.78, p=0.055) | 1.43 (0.72-2.82, p=0.307) |
| Lymph Nodes Examined | Mean (SD) | 1.00 (0.99-1.01, p=0.772) | 0.99 (0.98-1.00, p=0.008) |
| Margin Status | R0 | - | - |
|  | R1 | 6.12 (3.97-9.44, p<0.001) | 2.46 (1.47-4.13, p=0.001) |
| Lymphatic Involvement | No | - | - |
|  | Yes | 2.93 (2.46-3.49, p<0.001) | 1.44 (1.15-1.80, p=0.002) |
| Venous Involvement | No | - | - |
|  | Yes | 2.49 (2.10-2.95, p<0.001) | 1.04 (0.84-1.30, p=0.719) |
| Perineural Involvement | No | - | - |
|  | Yes | 3.16 (2.66-3.77, p<0.001) | 1.52 (1.22-1.90, p<0.001) |
| Extracapsular Spread | No | - | - |
|  | Yes | 2.63 (2.14-3.23, p<0.001) | 1.53 (1.19-1.98, p=0.001) |

Supplementary Table 3 Cox regression analyses on smoking status on recurrence-free survival after esophagectomy for esophageal cancer

|  |  | HR (univariable) | HR (multivariable) |
| --- | --- | --- | --- |
| Smoking Status | Current | - | - |
|  | Ex-Smoker | 0.90 (0.72-1.13, p=0.377) | 1.00 (0.78-1.29, p=0.980) |
|  | Never | 0.84 (0.65-1.08, p=0.173) | 1.03 (0.78-1.36, p=0.842) |
| Age at Diagnosis | Mean (SD) | 1.00 (0.99-1.01, p=0.836) | 0.99 (0.98-1.00, p=0.148) |
| Gender | Male | - | - |
|  | Female | 0.70 (0.56-0.88, p=0.002) | 0.65 (0.50-0.85, p=0.002) |
| Histology | Adenocarcinoma | - | - |
|  | SCC | 0.84 (0.67-1.05, p=0.127) | 1.34 (1.01-1.77, p=0.043) |
| Body Mass Index, kg/m2 | Mean (SD) | 0.98 (0.96-1.00, p=0.061) | 1.00 (0.98-1.02, p=0.909) |
| IMD Decile | 1 | - | - |
|  | 5 | 1.18 (0.89-1.57, p=0.256) | 1.37 (0.99-1.88, p=0.054) |
|  | 2 | 1.08 (0.82-1.42, p=0.571) | 1.09 (0.82-1.46, p=0.545) |
|  | 3 | 0.88 (0.65-1.19, p=0.417) | 1.25 (0.90-1.71, p=0.179) |
|  | 4 | 0.81 (0.58-1.11, p=0.188) | 0.87 (0.62-1.23, p=0.427) |
|  | Unknown | 2.83 (1.80-4.46, p<0.001) | 3.31 (1.99-5.50, p<0.001) |
| ASA Grade | Grade 1 | - | - |
|  | Grade 2 | 0.89 (0.69-1.16, p=0.392) | 0.89 (0.67-1.18, p=0.413) |
|  | Grade 3 | 1.08 (0.81-1.44, p=0.589) | 0.95 (0.70-1.31, p=0.774) |
|  | Grade 4 | 0.31 (0.04-2.20, p=0.239) | 0.25 (0.03-1.86, p=0.177) |
|  | Unknown | 1.30 (0.91-1.85, p=0.152) | 1.20 (0.80-1.81, p=0.371) |
| Overall Treatment | NAC + Surgery | - | - |
|  | Surgery Only | 0.91 (0.76-1.10, p=0.344) | 1.25 (1.01-1.55, p=0.041) |
| AJCC Pathological Stage Classification | Stage 0 | - | - |
|  | Stage I | 1.09 (0.53-2.25, p=0.811) | 1.28 (0.52-3.14, p=0.592) |
|  | Stage II | 1.76 (0.87-3.54, p=0.115) | 1.36 (0.56-3.29, p=0.491) |
|  | Stage III | 7.18 (3.70-13.95, p<0.001) | 4.40 (1.85-10.44, p=0.001) |
|  | Stage IVA | 9.96 (4.92-20.15, p<0.001) | 5.25 (2.13-12.94, p<0.001) |
| Tumor Grade | Well | - | - |
|  | Moderate | 4.11 (2.35-7.20, p<0.001) | 2.28 (1.25-4.15, p=0.007) |
|  | Poor | 6.44 (3.67-11.30, p<0.001) | 2.75 (1.50-5.07, p=0.001) |
|  | Unknown | 2.10 (1.05-4.23, p=0.037) | 1.95 (0.86-4.45, p=0.112) |
| Lymph Nodes Examined | Mean (SD) | 1.00 (1.00-1.01, p=0.242) | 0.99 (0.98-1.00, p=0.071) |
| Margin Status | R0 | - | - |
|  | R1 | 3.66 (2.05-6.53, p<0.001) | 1.22 (0.59-2.51, p=0.594) |
| Lymphatic Involvement | No | - | - |
|  | Yes | 3.06 (2.52-3.70, p<0.001) | 1.45 (1.14-1.84, p=0.003) |
| Venous Involvement | No | - | - |
|  | Yes | 2.51 (2.08-3.01, p<0.001) | 1.03 (0.82-1.30, p=0.772) |
| Perineural Involvement | No | - | - |
|  | Yes | 3.49 (2.88-4.23, p<0.001) | 1.61 (1.27-2.05, p<0.001) |
| Extracapsular Spread | No | - | - |
|  | Yes | 2.84 (2.29-3.53, p<0.001) | 1.57 (1.21-2.04, p=0.001) |

Supplementary Table 4 Baseline demographics and postoperative outcomes of

patients receiving neoadjuvant therapy and esophagectomy for esophageal cancer

stratified by smoking status

|  |  | Current,  n=145 | Ex-Smoker,  n=282 | Never,  n=174 | Total,  n=601 | p-value |
| --- | --- | --- | --- | --- | --- | --- |
| Age at Diagnosis | Median (IQR) | 60.0 (11.0) | 65.0 (10.0) | 65.0 (13.0) | 64.0 (12.0) | <0.001 |
| Gender | Male | 110 (75.9) | 235 (83.3) | 121 (69.5) | 466 (77.5) | 0.002 |
|  | Female | 35 (24.1) | 47 (16.7) | 53 (30.5) | 135 (22.5) |  |
| Histology | Adenocarcinoma | 112 (77.2) | 226 (80.1) | 129 (74.1) | 467 (77.7) | 0.323 |
|  | SCC | 33 (22.8) | 56 (19.9) | 45 (25.9) | 134 (22.3) |  |
| Body Mass Index, kg/m2 | Median (IQR) | 25.5 (7.4) | 27.1 (5.4) | 25.7 (6.0) | 26.4 (6.0) | 0.003 |
| IMD Decile | 1 | 48 (33.1) | 61 (21.6) | 17 (9.8) | 126 (21.0) | <0.001 |
|  | 2 | 36 (24.8) | 64 (22.7) | 42 (24.1) | 142 (23.6) |  |
|  | 3 | 22 (15.2) | 64 (22.7) | 33 (19.0) | 119 (19.8) |  |
|  | 4 | 17 (11.7) | 39 (13.8) | 33 (19.0) | 89 (14.8) |  |
|  | 5 | 14 (9.7) | 43 (15.2) | 44 (25.3) | 101 (16.8) |  |
|  | Unknown | 8 (5.5) | 11 (3.9) | 5 (2.9) | 24 (4.0) |  |
| ASA Grade | Grade 1 | 13 (9.0) | 39 (13.8) | 34 (19.5) | 86 (14.3) | 0.027 |
|  | Grade 2 | 75 (51.7) | 159 (56.4) | 95 (54.6) | 329 (54.7) |  |
|  | Grade 3 | 43 (29.7) | 69 (24.5) | 37 (21.3) | 149 (24.8) |  |
|  | Grade 4 | 2 (1.4) | 0 (0.0) | 0 (0.0) | 2 (0.3) |  |
|  | Unknown | 12 (8.3) | 15 (5.3) | 8 (4.6) | 35 (5.8) |  |
| AJCC Pathological Stage Classification | Stage 0 | 14 (9.7) | 21 (7.4) | 12 (6.9) | 47 (7.8) | 0.377 |
|  | Stage I | 22 (15.2) | 36 (12.8) | 16 (9.2) | 74 (12.3) |  |
|  | Stage II | 28 (19.3) | 74 (26.2) | 56 (32.2) | 158 (26.3) |  |
|  | Stage III | 64 (44.1) | 121 (42.9) | 73 (42.0) | 258 (42.9) |  |
|  | Stage IVA | 17 (11.7) | 30 (10.6) | 17 (9.8) | 64 (10.6) |  |
| Tumor Grade | Well | 9 (6.2) | 5 (1.8) | 6 (3.4) | 20 (3.3) | 0.084 |
|  | Moderate | 57 (39.3) | 144 (51.1) | 81 (46.6) | 282 (46.9) |  |
|  | Poor | 66 (45.5) | 107 (37.9) | 66 (37.9) | 239 (39.8) |  |
|  | Unknown | 13 (9.0) | 26 (9.2) | 21 (12.1) | 60 (10.0) |  |
| Lymph Nodes Examined | Median (IQR) | 35.0 (15.0) | 32.0 (15.0) | 31.0 (14.0) | 33.0 (15.0) | 0.051 |
| Margin Status | R0 | 142 (97.9) | 280 (99.3) | 173 (99.4) | 595 (99.0) | 0.327 |
|  | R1 | 3 (2.1) | 2 (0.7) | 1 (0.6) | 6 (1.0) |  |
| Lymphatic Involvement | No | 72 (49.7) | 145 (51.4) | 93 (53.4) | 310 (51.6) | 0.794 |
|  | Yes | 73 (50.3) | 137 (48.6) | 81 (46.6) | 291 (48.4) |  |
| Venous Involvement | No | 90 (62.1) | 176 (62.4) | 121 (69.5) | 387 (64.4) | 0.242 |
|  | Yes | 55 (37.9) | 106 (37.6) | 53 (30.5) | 214 (35.6) |  |
| Perineural Involvement | No | 75 (51.7) | 154 (54.6) | 97 (55.7) | 326 (54.2) | 0.762 |
|  | Yes | 70 (48.3) | 128 (45.4) | 77 (44.3) | 275 (45.8) |  |
| Extracapsular Spread | No | 116 (80.0) | 217 (77.0) | 130 (74.7) | 463 (77.0) | 0.535 |
|  | Yes | 29 (20.0) | 65 (23.0) | 44 (25.3) | 138 (23.0) |  |
| Critical Care Stay | Median (IQR) | 3.0 (5.0) | 2.0 (3.0) | 2.0 (3.0) | 2.0 (3.8) | <0.001 |
| Length of Stay | Median (IQR) | 14.5 (10.0) | 15.0 (9.0) | 14.0 (8.0) | 15.0 (9.0) | 0.814 |
| Overall Complications | No | 40 (27.6) | 103 (36.5) | 76 (43.7) | 219 (36.4) | 0.012 |
|  | Yes | 105 (72.4) | 179 (63.5) | 98 (56.3) | 382 (63.6) |  |
| Surgical Site Infection | No | 130 (89.7) | 257 (91.1) | 161 (92.5) | 548 (91.2) | 0.666 |
|  | Yes | 15 (10.3) | 25 (8.9) | 13 (7.5) | 53 (8.8) |  |
| Pulmonary Complications | No | 123 (84.8) | 243 (86.2) | 156 (89.7) | 522 (86.9) | 0.400 |
|  | Yes | 22 (15.2) | 39 (13.8) | 18 (10.3) | 79 (13.1) |  |
| Cardiac Complications | No | 135 (93.1) | 253 (89.7) | 156 (89.7) | 544 (90.5) | 0.474 |
|  | Yes | 10 (6.9) | 29 (10.3) | 18 (10.3) | 57 (9.5) |  |
| Anastomotic Leaks | No | 127 (87.6) | 262 (92.9) | 164 (94.3) | 553 (92.0) | 0.069 |
|  | Yes | 18 (12.4) | 20 (7.1) | 10 (5.7) | 48 (8.0) |  |
| In-hospital Mortality | No | 138 (95.2) | 276 (97.9) | 172 (98.9) | 586 (97.5) | 0.096 |
|  | Yes | 7 (4.8) | 6 (2.1) | 2 (1.1) | 15 (2.5) |  |
| 30-day Mortality | No | 141 (97.2) | 277 (98.2) | 173 (99.4) | 591 (98.3) | 0.310 |
|  | Yes | 4 (2.8) | 5 (1.8) | 1 (0.6) | 10 (1.7) |  |
|  |  |  |  |  |  |  |

Supplementary Table 5 Baseline demographics and postoperative outcomes of

patients receiving esophagectomy only for esophageal cancer stratified by smoking

status

|  |  | Current,  n=137 | Ex-Smoker,  n=248 | Never,  n=182 | Total,  n=567 | p-value |
| --- | --- | --- | --- | --- | --- | --- |
| Age at Diagnosis | Median (IQR) | 63.0 (15.0) | 68.0 (11.0) | 67.5 (15.5) | 67.0 (13.0) | <0.001 |
| Gender | Male | 94 (68.6) | 195 (78.6) | 107 (58.8) | 396 (69.8) | <0.001 |
|  | Female | 43 (31.4) | 53 (21.4) | 75 (41.2) | 171 (30.2) |  |
| Histology | Adenocarcinoma | 93 (67.9) | 202 (81.5) | 129 (70.9) | 424 (74.8) | 0.005 |
|  | SCC | 44 (32.1) | 46 (18.5) | 53 (29.1) | 143 (25.2) |  |
| Body Mass Index, kg/m2 | Median (IQR) | 23.0 (6.0) | 26.1 (5.8) | 26.2 (5.6) | 25.6 (6.2) | <0.001 |
| IMD Decile | 1 | 38 (27.7) | 61 (24.6) | 34 (18.7) | 133 (23.5) | 0.001 |
|  | 2 | 41 (29.9) | 49 (19.8) | 33 (18.1) | 123 (21.7) |  |
|  | 3 | 23 (16.8) | 46 (18.5) | 32 (17.6) | 101 (17.8) |  |
|  | 4 | 18 (13.1) | 41 (16.5) | 26 (14.3) | 85 (15.0) |  |
|  | 5 | 12 (8.8) | 41 (16.5) | 54 (29.7) | 107 (18.9) |  |
|  | Unknown | 5 (3.6) | 10 (4.0) | 3 (1.6) | 18 (3.2) |  |
| ASA Grade | Grade 1 | 20 (14.6) | 31 (12.5) | 38 (20.9) | 89 (15.7) | 0.030 |
|  | Grade 2 | 50 (36.5) | 117 (47.2) | 85 (46.7) | 252 (44.4) |  |
|  | Grade 3 | 49 (35.8) | 65 (26.2) | 33 (18.1) | 147 (25.9) |  |
|  | Grade 4 | 1 (0.7) | 3 (1.2) | 2 (1.1) | 6 (1.1) |  |
|  | Unknown | 17 (12.4) | 32 (12.9) | 24 (13.2) | 73 (12.9) |  |
| AJCC Pathological Stage Classification | Stage 0 | 2 (1.5) | 13 (5.2) | 11 (6.0) | 26 (4.6) | 0.052 |
|  | Stage I | 31 (22.6) | 78 (31.5) | 65 (35.7) | 174 (30.7) |  |
|  | Stage II | 21 (15.3) | 44 (17.7) | 30 (16.5) | 95 (16.8) |  |
|  | Stage III | 70 (51.1) | 98 (39.5) | 66 (36.3) | 234 (41.3) |  |
|  | Stage IVA | 13 (9.5) | 15 (6.0) | 10 (5.5) | 38 (6.7) |  |
| Tumor Grade | Well | 17 (12.4) | 34 (13.7) | 30 (16.5) | 81 (14.3) | 0.474 |
|  | Moderate | 70 (51.1) | 120 (48.4) | 84 (46.2) | 274 (48.3) |  |
|  | Poor | 45 (32.8) | 80 (32.3) | 52 (28.6) | 177 (31.2) |  |
|  | Unknown | 5 (3.6) | 14 (5.6) | 16 (8.8) | 35 (6.2) |  |
| Lymph Nodes Examined | Median (IQR) | 26.0 (16.2) | 26.0 (13.0) | 29.0 (13.2) | 27.0 (15.0) | 0.034 |
| Margin Status | R0 | 130 (94.9) | 244 (98.4) | 174 (95.6) | 548 (96.6) | 0.120 |
|  | R1 | 7 (5.1) | 4 (1.6) | 8 (4.4) | 19 (3.4) |  |
| Lymphatic Involvement | No | 77 (56.2) | 144 (58.1) | 110 (60.4) | 331 (58.4) | 0.743 |
|  | Yes | 60 (43.8) | 104 (41.9) | 72 (39.6) | 236 (41.6) |  |
| Venous Involvement | No | 91 (66.4) | 161 (64.9) | 125 (68.7) | 377 (66.5) | 0.716 |
|  | Yes | 46 (33.6) | 87 (35.1) | 57 (31.3) | 190 (33.5) |  |
| Perineural Involvement | No | 69 (50.4) | 142 (57.3) | 115 (63.2) | 326 (57.5) | 0.072 |
|  | Yes | 68 (49.6) | 106 (42.7) | 67 (36.8) | 241 (42.5) |  |
| Extracapsular Spread | No | 126 (92.0) | 228 (91.9) | 166 (91.2) | 520 (91.7) | 0.956 |
|  | Yes | 11 (8.0) | 20 (8.1) | 16 (8.8) | 47 (8.3) |  |
| Critical Care Stay | Median (IQR) | 3.0 (8.0) | 3.0 (5.0) | 2.0 (3.0) | 3.0 (5.0) | 0.137 |
| Length of Stay | Median (IQR) | 17.0 (13.0) | 17.0 (14.0) | 16.0 (10.0) | 16.0 (13.0) | 0.473 |
| Overall Complications | No | 36 (26.3) | 78 (31.5) | 60 (33.0) | 174 (30.7) | 0.414 |
|  | Yes | 101 (73.7) | 170 (68.5) | 122 (67.0) | 393 (69.3) |  |
| Surgical Site Infection | No | 121 (88.3) | 220 (88.7) | 164 (90.1) | 505 (89.1) | 0.855 |
|  | Yes | 16 (11.7) | 28 (11.3) | 18 (9.9) | 62 (10.9) |  |
| Pulmonary Complications | No | 121 (88.3) | 228 (91.9) | 165 (90.7) | 514 (90.7) | 0.506 |
|  | Yes | 16 (11.7) | 20 (8.1) | 17 (9.3) | 53 (9.3) |  |
| Cardiac Complications | No | 134 (97.8) | 241 (97.2) | 172 (94.5) | 547 (96.5) | 0.207 |
|  | Yes | 3 (2.2) | 7 (2.8) | 10 (5.5) | 20 (3.5) |  |
| Anastomotic Leaks | No | 126 (92.0) | 221 (89.1) | 170 (93.4) | 517 (91.2) | 0.280 |
|  | Yes | 11 (8.0) | 27 (10.9) | 12 (6.6) | 50 (8.8) |  |
| In-hospital Mortality | No | 129 (94.2) | 240 (96.8) | 170 (93.4) | 539 (95.1) | 0.241 |
|  | Yes | 8 (5.8) | 8 (3.2) | 12 (6.6) | 28 (4.9) |  |
| 30-day Mortality | No | 132 (96.4) | 243 (98.0) | 171 (94.0) | 546 (96.3) | 0.092 |
|  | Yes | 5 (3.6) | 5 (2.0) | 11 (6.0) | 21 (3.7) |  |

Supplementary Table 6 Baseline demographics and postoperative outcomes of

patients receiving transthoracic esophagectomy for esophageal adenocarcinoma

stratified by smoking status

|  |  | Current | Ex-Smoker | Never | Total | p-value |
| --- | --- | --- | --- | --- | --- | --- |
| Age at Diagnosis | Median (IQR) | 62.0 (14.0) | 66.0 (12.0) | 66.0 (13.8) | 65.0 (12.5) | <0.001 |
| Gender | Male | 163 (79.5) | 380 (88.8) | 206 (79.8) | 749 (84.1) | 0.001 |
|  | Female | 42 (20.5) | 48 (11.2) | 52 (20.2) | 142 (15.9) |  |
| Body Mass Index, kg/m2 | Median (IQR) | 25.5 (7.5) | 27.1 (5.3) | 26.8 (5.8) | 26.7 (6.0) | <0.001 |
| IMD Decile | 1 | 63 (30.7) | 100 (23.4) | 41 (15.9) | 204 (22.9) | <0.001 |
|  | 2 | 58 (28.3) | 85 (19.9) | 52 (20.2) | 195 (21.9) |  |
|  | 3 | 34 (16.6) | 91 (21.3) | 49 (19.0) | 174 (19.5) |  |
|  | 4 | 25 (12.2) | 62 (14.5) | 38 (14.7) | 125 (14.0) |  |
|  | 5 | 14 (6.8) | 69 (16.1) | 73 (28.3) | 156 (17.5) |  |
|  | Unknown | 11 (5.4) | 21 (4.9) | 5 (1.9) | 37 (4.2) |  |
| ASA Grade | Grade 1 | 26 (12.7) | 54 (12.6) | 55 (21.3) | 135 (15.2) | 0.001 |
|  | Grade 2 | 91 (44.4) | 224 (52.3) | 136 (52.7) | 451 (50.6) |  |
|  | Grade 3 | 70 (34.1) | 107 (25.0) | 42 (16.3) | 219 (24.6) |  |
|  | Grade 4 | 2 (1.0) | 2 (0.5) | 2 (0.8) | 6 (0.7) |  |
|  | Unknown | 16 (7.8) | 41 (9.6) | 23 (8.9) | 80 (9.0) |  |
| Overall Treatmet | NAC + Surgery | 112 (54.6) | 226 (52.8) | 129 (50.0) | 467 (52.4) | 0.596 |
|  | Surgery Only | 93 (45.4) | 202 (47.2) | 129 (50.0) | 424 (47.6) |  |
| AJCC Pathological Stage Classification | Stage 0 | 9 (4.4) | 22 (5.1) | 16 (6.2) | 47 (5.3) | 0.762 |
|  | Stage I | 45 (22.0) | 102 (23.8) | 64 (24.8) | 211 (23.7) |  |
|  | Stage II | 31 (15.1) | 85 (19.9) | 50 (19.4) | 166 (18.6) |  |
|  | Stage III | 98 (47.8) | 180 (42.1) | 106 (41.1) | 384 (43.1) |  |
|  | Stage IVA | 22 (10.7) | 39 (9.1) | 22 (8.5) | 83 (9.3) |  |
| Tumor Grade | Well | 20 (9.8) | 29 (6.8) | 30 (11.6) | 79 (8.9) | 0.122 |
|  | Moderate | 85 (41.5) | 211 (49.3) | 110 (42.6) | 406 (45.6) |  |
|  | Poor | 89 (43.4) | 163 (38.1) | 97 (37.6) | 349 (39.2) |  |
|  | Unknown | 11 (5.4) | 25 (5.8) | 21 (8.1) | 57 (6.4) |  |
| Lymph Nodes Examined | Median (IQR) | 32.0 (17.0) | 30.0 (16.0) | 31.0 (13.0) | 31.0 (16.0) | 0.277 |
| Margin Status | R0 | 199 (97.1) | 422 (98.6) | 249 (96.5) | 870 (97.6) | 0.181 |
|  | R1 | 6 (2.9) | 6 (1.4) | 9 (3.5) | 21 (2.4) |  |
| Lymphatic Involvement | No | 103 (50.2) | 226 (52.8) | 129 (50.0) | 458 (51.4) | 0.723 |
|  | Yes | 102 (49.8) | 202 (47.2) | 129 (50.0) | 433 (48.6) |  |
| Venous Involvement | No | 127 (62.0) | 266 (62.1) | 175 (67.8) | 568 (63.7) | 0.270 |
|  | Yes | 78 (38.0) | 162 (37.9) | 83 (32.2) | 323 (36.3) |  |
| Perineural Involvement | No | 99 (48.3) | 230 (53.7) | 143 (55.4) | 472 (53.0) | 0.283 |
|  | Yes | 106 (51.7) | 198 (46.3) | 115 (44.6) | 419 (47.0) |  |
| Extracapsular Spread | No | 172 (83.9) | 359 (83.9) | 207 (80.2) | 738 (82.8) | 0.423 |
|  | Yes | 33 (16.1) | 69 (16.1) | 51 (19.8) | 153 (17.2) |  |
| Critical Care Stay | Median (IQR) | 3.0 (7.0) | 2.0 (3.0) | 2.0 (3.0) | 2.0 (4.0) | 0.010 |
| Length of Stay | Median (IQR) | 14.0 (10.0) | 15.0 (10.0) | 15.0 (8.0) | 15.0 (10.0) | 0.517 |
| Overall Complications | No | 60 (29.3) | 145 (33.9) | 101 (39.1) | 306 (34.3) | 0.081 |
|  | Yes | 145 (70.7) | 283 (66.1) | 157 (60.9) | 585 (65.7) |  |
| Surgical site infection | No | 183 (89.3) | 380 (88.8) | 234 (90.7) | 797 (89.5) | 0.729 |
|  | Yes | 22 (10.7) | 48 (11.2) | 24 (9.3) | 94 (10.5) |  |
| Pulmonary Complications | No | 180 (87.8) | 380 (88.8) | 230 (89.1) | 790 (88.7) | 0.897 |
|  | Yes | 25 (12.2) | 48 (11.2) | 28 (10.9) | 101 (11.3) |  |
| Cardiac Complications | No | 198 (96.6) | 399 (93.2) | 237 (91.9) | 834 (93.6) | 0.108 |
|  | Yes | 7 (3.4) | 29 (6.8) | 21 (8.1) | 57 (6.4) |  |
| Anastomotic Leaks | No | 185 (90.2) | 391 (91.4) | 242 (93.8) | 818 (91.8) | 0.343 |
|  | Yes | 20 (9.8) | 37 (8.6) | 16 (6.2) | 73 (8.2) |  |
| In-Hospital Mortality | No | 195 (95.1) | 419 (97.9) | 250 (96.9) | 864 (97.0) | 0.162 |
|  | Yes | 10 (4.9) | 9 (2.1) | 8 (3.1) | 27 (3.0) |  |
| 30-day Mortality | No | 198 (96.6) | 422 (98.6) | 251 (97.3) | 871 (97.8) | 0.232 |
|  | Yes | 7 (3.4) | 6 (1.4) | 7 (2.7) | 20 (2.2) |  |

Supplementary Table 7 Baseline demographics and postoperative outcomes of

patients receiving transthoracic esophagectomy for esophageal adenocarcinoma

stratified by smoking status

|  |  | Current | Ex-Smoker | Never | Total | p-value |
| --- | --- | --- | --- | --- | --- | --- |
| Age at Diagnosis | Median (IQR) | 61.0 (15.0) | 67.0 (9.8) | 69.0 (11.8) | 66.0 (12.0) | <0.001 |
| Gender | Male | 41 (53.2) | 50 (49.0) | 22 (22.4) | 113 (40.8) | <0.001 |
|  | Female | 36 (46.8) | 52 (51.0) | 76 (77.6) | 164 (59.2) |  |
| Body Mass Index, kg/m2 | Median (IQR) | 22.0 (5.5) | 24.3 (5.6) | 24.1 (5.4) | 23.7 (5.6) | 0.006 |
| IMD Decile | 1 | 23 (29.9) | 22 (21.6) | 10 (10.2) | 55 (19.9) | 0.057 |
|  | 2 | 19 (24.7) | 28 (27.5) | 23 (23.5) | 70 (25.3) |  |
|  | 3 | 11 (14.3) | 19 (18.6) | 16 (16.3) | 46 (16.6) |  |
|  | 4 | 10 (13.0) | 18 (17.6) | 21 (21.4) | 49 (17.7) |  |
|  | 5 | 12 (15.6) | 15 (14.7) | 25 (25.5) | 52 (18.8) |  |
|  | Unknown | 2 (2.6) | 0 (0.0) | 3 (3.1) | 5 (1.8) |  |
| ASA Grade | Grade 1 | 7 (9.1) | 16 (15.7) | 17 (17.3) | 40 (14.4) | 0.308 |
|  | Grade 2 | 34 (44.2) | 52 (51.0) | 44 (44.9) | 130 (46.9) |  |
|  | Grade 3 | 22 (28.6) | 27 (26.5) | 28 (28.6) | 77 (27.8) |  |
|  | Grade 4 | 1 (1.3) | 1 (1.0) | 0 (0.0) | 2 (0.7) |  |
|  | Unknown | 13 (16.9) | 6 (5.9) | 9 (9.2) | 28 (10.1) |  |
| Overall Treatment | NAC + Surgery | 33 (42.9) | 56 (54.9) | 45 (45.9) | 134 (48.4) | 0.233 |
|  | Surgery Only | 44 (57.1) | 46 (45.1) | 53 (54.1) | 143 (51.6) |  |
| AJCC Pathological Stage Classification | Stage 0 | 7 (9.1) | 12 (11.8) | 7 (7.1) | 26 (9.4) | 0.309 |
|  | Stage I | 8 (10.4) | 12 (11.8) | 17 (17.3) | 37 (13.4) |  |
|  | Stage II | 18 (23.4) | 33 (32.4) | 36 (36.7) | 87 (31.4) |  |
|  | Stage III | 36 (46.8) | 39 (38.2) | 33 (33.7) | 108 (39.0) |  |
|  | Stage IVA | 8 (10.4) | 6 (5.9) | 5 (5.1) | 19 (6.9) |  |
| Tumor Grade | Well | 6 (7.8) | 10 (9.8) | 6 (6.1) | 22 (7.9) | 0.715 |
|  | Moderate | 42 (54.5) | 53 (52.0) | 55 (56.1) | 150 (54.2) |  |
|  | Poor | 22 (28.6) | 24 (23.5) | 21 (21.4) | 67 (24.2) |  |
|  | Unknown | 7 (9.1) | 15 (14.7) | 16 (16.3) | 38 (13.7) |  |
| Lymph Nodes Examined | Median (IQR) | 27.5 (18.0) | 27.0 (10.0) | 29.0 (14.0) | 28.0 (14.0) | 0.136 |
| Margin Status | R0 | 73 (94.8) | 102 (100.0) | 98 (100.0) | 273 (98.6) | 0.005 |
|  | R1 | 4 (5.2) | 0 (0.0) | 0 (0.0) | 4 (1.4) |  |
| Lymphatic Involvement | No | 46 (59.7) | 63 (61.8) | 74 (75.5) | 183 (66.1) | 0.047 |
|  | Yes | 31 (40.3) | 39 (38.2) | 24 (24.5) | 94 (33.9) |  |
| Venous Involvement | No | 54 (70.1) | 71 (69.6) | 71 (72.4) | 196 (70.8) | 0.898 |
|  | Yes | 23 (29.9) | 31 (30.4) | 27 (27.6) | 81 (29.2) |  |
| Perineural Involvement | No | 45 (58.4) | 66 (64.7) | 69 (70.4) | 180 (65.0) | 0.257 |
|  | Yes | 32 (41.6) | 36 (35.3) | 29 (29.6) | 97 (35.0) |  |
| Extracapsular Spread | No | 70 (90.9) | 86 (84.3) | 89 (90.8) | 245 (88.4) | 0.259 |
|  | Yes | 7 (9.1) | 16 (15.7) | 9 (9.2) | 32 (11.6) |  |
| Critical Care Stay | Median (IQR) | 3.0 (7.5) | 2.0 (5.0) | 2.0 (3.2) | 3.0 (5.0) | 0.026 |
| Length of Stay | Median (IQR) | 18.0 (14.5) | 15.0 (12.8) | 15.0 (10.2) | 16.0 (13.2) | 0.055 |
| Overall Complications | No | 16 (20.8) | 36 (35.3) | 35 (35.7) | 87 (31.4) | 0.061 |
|  | Yes | 61 (79.2) | 66 (64.7) | 63 (64.3) | 190 (68.6) |  |
| Surgical site infection | No | 68 (88.3) | 97 (95.1) | 91 (92.9) | 256 (92.4) | 0.232 |
|  | Yes | 9 (11.7) | 5 (4.9) | 7 (7.1) | 21 (7.6) |  |
| Pulmonary Complications | No | 64 (83.1) | 91 (89.2) | 91 (92.9) | 246 (88.8) | 0.126 |
|  | Yes | 13 (16.9) | 11 (10.8) | 7 (7.1) | 31 (11.2) |  |
| Cardiac Complications | No | 71 (92.2) | 95 (93.1) | 91 (92.9) | 257 (92.8) | 0.971 |
|  | Yes | 6 (7.8) | 7 (6.9) | 7 (7.1) | 20 (7.2) |  |
| Anastomotic Leaks | No | 68 (88.3) | 92 (90.2) | 92 (93.9) | 252 (91.0) | 0.418 |
|  | Yes | 9 (11.7) | 10 (9.8) | 6 (6.1) | 25 (9.0) |  |
| In-Hospital Mortality | No | 72 (93.5) | 97 (95.1) | 92 (93.9) | 261 (94.2) | 0.888 |
|  | Yes | 5 (6.5) | 5 (4.9) | 6 (6.1) | 16 (5.8) |  |
| 30-day Mortality | No | 75 (97.4) | 98 (96.1) | 93 (94.9) | 266 (96.0) | 0.701 |
|  | Yes | 2 (2.6) | 4 (3.9) | 5 (5.1) | 11 (4.0) |  |

Supplementary Figure 1 Trends over time on rates of Ivor-Lewis esophagectomy, neoadjuvant therapy, overall complications and anastomotic leaks

Supplementary Figure 2 Impact of smoking in patients undergoing esophagectomy for esophageal adenocarcinoma on survival (A) Overall survival (B) Cancer-specific survival (C) Recurrence-free survival

**A**

**B**

**C**

**Overall Survival**

**Recurrence-free Survival**

**Cancer-specific Survival**

Supplementary Figure 3 Impact of smoking in patients undergoing esophagectomy for esophageal squamous cell carcinoma on survival (A) Overall survival (B) Cancer-specific survival (C) Recurrence-free survival

**A**

**B**

**C**

**Overall Survival**

**Recurrence-free Survival**

**Cancer-specific Survival**

p<0.001

p=0.040

p=0.4
